# Multimorbidity Patterns and the Disablement Process among Public Long-Term Care Insurance Claimants in the City of Yiwu (Zhejiang Province, China)

**DOI:** 10.3390/ijerph19020645

**Published:** 2022-01-06

**Authors:** Chundi Liu, Renfang Shu, Hong Liang, Yan Liang

**Affiliations:** 1 School of Nursing, Fudan University, Shanghai 200032, China; 19301170066@fudan.edu.cn (C.L.); 19301170084@fudan.edu.cn (R.S.); 2 School of Social Development and Public Policy, Fudan University, Shanghai 200433, China; lianghong@fudan.edu.cn

**Keywords:** multimorbidity, patterns, disability, older adults, disablement process model

## Abstract

This study aimed to identify multimorbidity patterns and explore the disablement process by utilizing the model raised by Verbrugge and Jette as a theoretical framework. This cross-sectional study used public Long-term Care Insurance (LTCI) claimants’ assessment data of Yiwu city in Zhejiang Province, China, for 2604 individuals aged 60 years and older, from September through December 2018. Latent Class Analysis (LCA) was conducted using 10 common chronic conditions. Structural Equation Modeling was used to examine the disablement process. The latent classes of multimorbidity patterns were the “coronary atherosclerotic heart disease” class (19.0%), the “lower limb fractures” class (26.4%), and the “other diseases” class (54.6%). The structural model results show that coronary atherosclerotic heart disease had a significant influence on incontinence, but it was not statistically significant in predicting vision impairment and mobility impairment. Lower limb fractures had significant effects on vision impairment, incontinence, and mobility impairment. Vision impairment, incontinence, and mobility impairment had significant effects on physical activities of daily living (ADLs). Our findings suggest that different impairments exist from specific patterns of multimorbidity to physical ADL disability, which may provide insights for researchers and policy makers to develop tailored care and provide support for physically disabled older people.

## 1. Introduction

The world population is aging rapidly in both developing and developed countries. Moreover, the risk of disability increases with increasing age [1]. Disability not only affects the population’s healthy aging, but also imposes a heavy burden on family and societies [2]. China has a large, aging population, and it has been predicted that by 2025, China’s population aged over 60 years with a disability (those who experience more than moderate household difficulties) will reach 140 million, and the disability rate in older adults will be 7.0% [3]. In response to this challenge, the Chinese government has worked to establish a national public long-term care insurance (LTCI) program. Since 2016, 15 cities in China have implemented an LTCI policy as the first batch of pilot cities [4]. Subsequently, in 2020, a second batch of pilot cities was included [5], reaching a total of 49 pilot cities between the two batches. The policy target population is mainly physical disabled older people in the current stage, but will expand to the intellectual disabled, and will cover all ages in the future.

Research on the disablement process among older adults is critical. Disability is a process rather than a static end state [6]. Examining the process of becoming disabled gives us an opportunity to take potential interventions at earlier stages, when people are in a less disabled state. The Disablement Process model [6] provides a conceptual framework to understand the main pathway that links pathology, impairments, functional limitations, and disability. Previous research has examined the components of this model in the oldest-old [7] and in older ethnic groups [8], but it has lacked evidence among potential physical disabled older people. Therefore, it will be helpful to examine the Disablement Process model among the target population of LTCI, potential physically disabled older people, to inform research and policy and develop more tailored preventive care and support for this vulnerable population in the transition from less disabled to more severely disabled.

Further, in previous research using the Disablement Process model, pathology has been measured by disease severity [7] or the number of chronic conditions [9]. However, these approaches may not adequately capture larger clusters of chronic disease or health conditions [10] linked to disability. With the increasing prevalence of multimorbidity, that is, the coexistence of two or more chronic diseases in the same individual, multimorbidity patterns [10,11,12,13] have been used in recent years to describe the qualitative combinations of conditions so as to aid in developing tailored interventions. Latent class analysis (LCA) is a statistical approach that groups individuals into distinct homogeneous subgroups based on a set of observed variables. Although LCA has been performed in the US general population [13], and in older adults in Spain [10], to evaluate multimorbidity, to our knowledge, it has not been applied in potential physically disabled older people. Further, how the multimorbidity patterns develop into disability has not been examined under the guidance of the Disablement Process model.

Thus, the present study aimed to identify multimorbidity patterns and explore the disablement process among potential physically disabled older people, using public LTCI claimants’ data of 2604 older people in the city of Yiwu, Zhejiang Province, China, with the Disablement Process model [6] as a theoretical framework. The Disablement Process model [6] is a widely used model in gerontology to describe the pathway for how chronic diseases can lead to disability. The primary components in the pathway included pathology, impairment, functional limitations, and disability [6]. The proposed main pathway in the Disablement Process model is that pathology influences impairments, which lead to functional limitations, which finally lead to disability [8]. In this study, pathology was measured by multimorbidity patterns using LCA. Impairments and functional limitations were combined and labeled as impairments and measured by vision impairment and incontinence and mobility impairment based on the definitions by Verbrugge and Jette [6], and on measurements in Hung and Ross’s research [14]. A further objective was to explore, through multi-group structural equation analysis, whether the relationships among the study variables differ among different age groups, as well as between men and women.

## 2. Materials and Methods

### 2.1. Data Sources and Participants

This was a cross-sectional design study. We obtained data for this study from the public Long-term Care Insurance (LTCI) database of Yiwu, Zhejiang province, China. Yiwu is located in the middle of Zhejiang Province. As the key contact city in China’s National Long-term Care Insurance Polit Project, Yiwu had a total of 1.07 million permanent residents insured as of 2018, and 10% of them were aged 60 and over. Yiwu LTCI was conducted in September 2018, and the target population was adults aged 60. A set of standardized assessments was administered by trained professionals who visited claimants’ homes or facilities to determine the qualification of being an LTCI beneficiary. Eligible older adults were 60 and over years of age, and received the qualification assessment between 1 September and 31 December 2018. We excluded older adults with incomplete assessment data. The study used deidentified data including information on the medical diagnosis, impairments, disability, and sociodemographic characteristics. In total, 2604 older people were included for analysis. The mean age of the sample was 79.7 (standard deviation, SD = 9.14), 53.4% (*n* = 1391) of the sample were female, and 74.3% (*n* = 1935) were married. 

### 2.2. Variables Description

Pathology. In the Disablement Process model [6], pathology refers to biochemical and physiological abnormalities that are medically labeled as disease or developmental conditions. In this study, pathology was operationalized as multimorbidity patterns. We used medical diagnosis information to reflect existing morbidity. Ten chronic conditions were selected based on prevalence and were included in LTCI qualification assessment, and the chronic conditions were certified by trained professionals (doctors) using claimants’ medical history, clinical examination, self-reported information, and proxy interviews. LCA was conducted to generate latent classes of multimorbidity patterns.

Impairments. Impairments are dysfunctions and significant structural abnormalities [6]. Impairments were measured from three aspects: vision impairment, incontinence, and mobility impairment. (1) Vision impairment was measured using one item to assess the overall vision status by professionals based on the participant’s self-reported information: “Which statement best describes your present vision?” The response categories were as follows: 1 = basically normal; 2 = a little vision impairment and some difficulty in activities of daily living; 3 = some vision impairment and severe difficulty in activities of daily living; 4 = severe impairment: only with a little visual perception (e.g., perception of the shape of a hand within 1 m); 5 = severe impairment: blindness or no visual perception. (2) Incontinence was assessed through two self-reported items: “Which statement best describes your present urinary incontinence/fecal incontinence?” The response categories were as follows: 1 = without incontinence; 2 = about once a month; 3 = about once a week; 4 = about once per day; 5 = almost every time. (3) Mobility was assessed using six items, including turning over in bed, from sitting to standing, sitting in a chair, walking 5 m, keeping balance, and maneuvering up and down stairs. Each activity was given a score corresponding to 1–5 levels of dependency, ranging from 1 (without assistance) to 5 (with full assistance).

Disability. Disability is experiencing difficulty doing activities in any domain of life due to a health or physical problem [6]. Disability was defined as dependence in physical ADLs. Eight items were used to assess the current level of a participant’s ability in eating, brushing the teeth, washing the face, grooming, wearing clothes, wearing pants, bathing, and toileting. Each activity was given a score corresponding to 1–5 levels of dependency, ranging from 1 (without assistance) to 5 (with fully assistance) (scales of impairment and disability assessment see Supplementary file S1).

CFA was performed to generate factor scores of incontinence, mobility impairment, and physical ADLs. The higher the score, the more severe the impairment or the greater the dependency.

Confounders. Age, sex, education, and marital status were considered as confounders and were self-reported as follows: (1) age (60–74 years, 75–84 years, and 85 years and older); (2) sex (female and male); (3) educational attainment (illiterate, primary school, and middle school and more); and (4) marital status (married and others).

### 2.3. Statistical Analysis

A series of LCA models ranging from two to four classes were conducted on the 2604 participants to determine the laten classes of multimorbidity patterns. We used the adjusted Bayesian Information Criterion (aBIC) [15] to determine the optimal number of latent classes, where the lowest values indicate the best fitting model. Entropy was used to summarize the overall precision of the classification for all samples across all latent classes. The values of entropy ranged from 0 to 1, with scores close to 1 indicating a clear classification [16]. The Lo–Mendell–Rubin (LMR) and bootstrapped likelihood ratio tests (BLRT) were also used to evaluate model improvement as the number of classes increased, with a significant *p* value indicating that the T-class model provides a better fit for the data than the T-1 class model [17]. Furthermore, interpretability and clinical judgment were used.

Structural Equation Modeling (SEM) was used to examine the disablement process. Confirmative factor analysis was used to verify the structure validity of latent variables measured by the indicators as described in the variable description section. Model fit was assessed using the following indexes: Comparative Fit Index (CFI) > 0.9, Tucker–Lewis Index (TLI) > 0.9, Root Mean Square Error of Approximation (RMSEA) < 0.08, Standardized Root Mean Square Residual (SRMR) < 0.05, and Chi-square/df < 5.0. A multi-group analysis in SEM was performed to explore the differences among different age groups, as well as between men and women. All analyses were performed with Mplus 8.0 and Stata SE 15.0.

## 3. Results

### 3.1. Participant Characteristics

Table 1 presents the participants’ characteristics. Among the 2604 older adults, 35.0% (n = 911) were 85 or over, 34.2% (n = 890) were between 75 and 84; 53.4% (n = 1391) were female; and 74.3% (n = 1935) were married. In terms of educational levels, 54.8% (n = 1426) were illiterate, and 28.2% (n = 736) had graduated from primary school; 28.0% had two or more chronic conditions with a doctor’s certificate.

### 3.2. Multimorbidity Patterns: A Three-Class Model

Table 2 shows the results from the LCA. The three-class model yielded the lowest adjusted BIC (aBIC = 15,763.588) and the highest entropy (entropy = 0.979). Meanwhile, *p* values were significant both for the LMR and bootstrapped likelihood ratio tests (BLRT), indicating that the-three class model provided a better fit to the data than the two-class model. Furthermore, the three-class model was interpretable and reasonably well defined (Table 3). Table 3 presents the proportion of 2604 Long-term Care Insurance claimant aged ≥ 60 years within each latent class assignment based on disease onset. The final latent classes were as follows: (1) older adults who had coronary atherosclerotic heart disease (100%) and had other diseases, such as lower limb fractures and chronic obstructive pulmonary disease (19.0% of subjects, “coronary atherosclerotic heart disease”); (2) older adults who had lower limb fractures (100%) and had other diseases, such as advanced cancer (26.4% of subjects, “lower limb fractures”); and (3) older adults who didn’t have coronary atherosclerotic heart disease and lower limb fractures but had other diseases, such as advanced cancer, cerebral infarction, and chronic obstructive pulmonary disease (54.6% of subjects, “other diseases”).

### 3.3. Measurement Model

CFA was performed to confirm an acceptable fit of the latent variable constructs: mobility, incontinence, and physical ADLs. CFA showed good fit indices for the three latent variables: Chi-square/df = 4.04, *p* < 0.001, CFI = 0.995, TLI = 0.991, RMSEA = 0.034, and SRMR = 0.018, after model re-specification by correlating error terms according to empirical rationales. The standardized factor loadings of the observed variables ranged from 0.743 to 0.957 (see Table 4).

### 3.4. Structural Model

**Figure 1:**
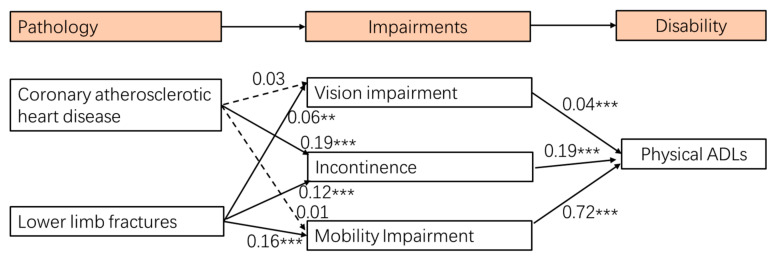
Structural model results. Notes: ** *p* < 0.01, *** *p* < 0.001; dashed lines represent non-significant pathways. Controlled age, sex, education, and marital status.

**Figure 2:**
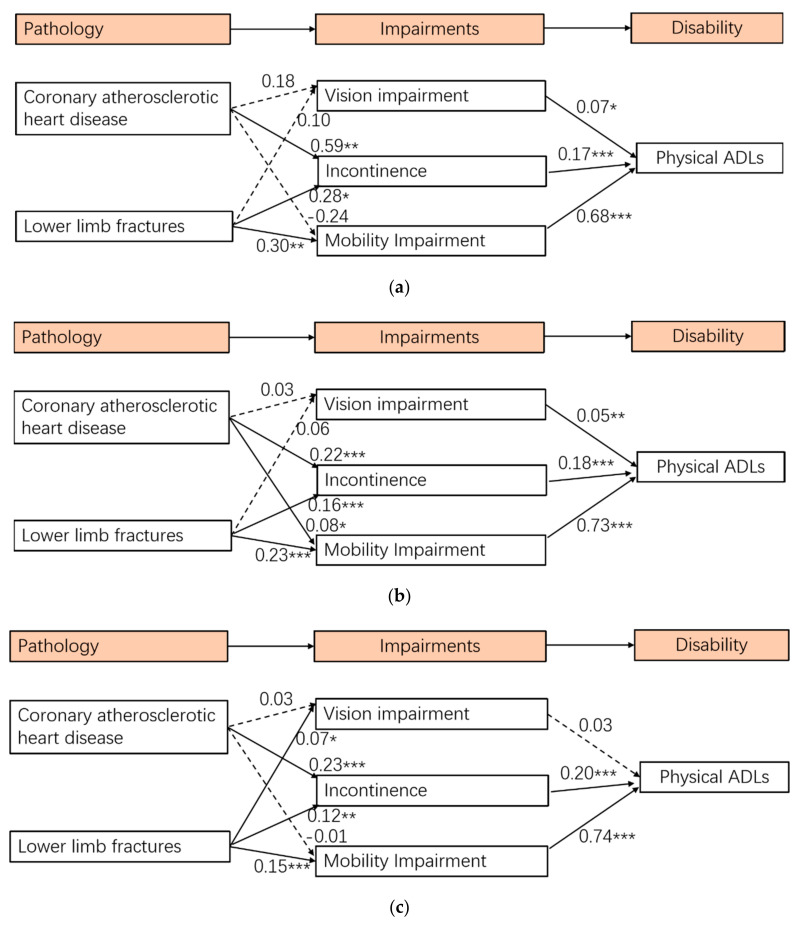
(**a**) Multigroup analysis in the SEM among older adults aged 60–74. (**b**) Multigroup analysis in the SEM among older adults aged 75–84. (**c**) Multigroup analysis in the SEM among older adults aged 85+. Notes: * *p* < 0.05, ** *p* < 0.01, *** *p* < 0.001; dashed lines represent non-significant pathways. Controlled sex, education, and marital status.

**Figure 3:**
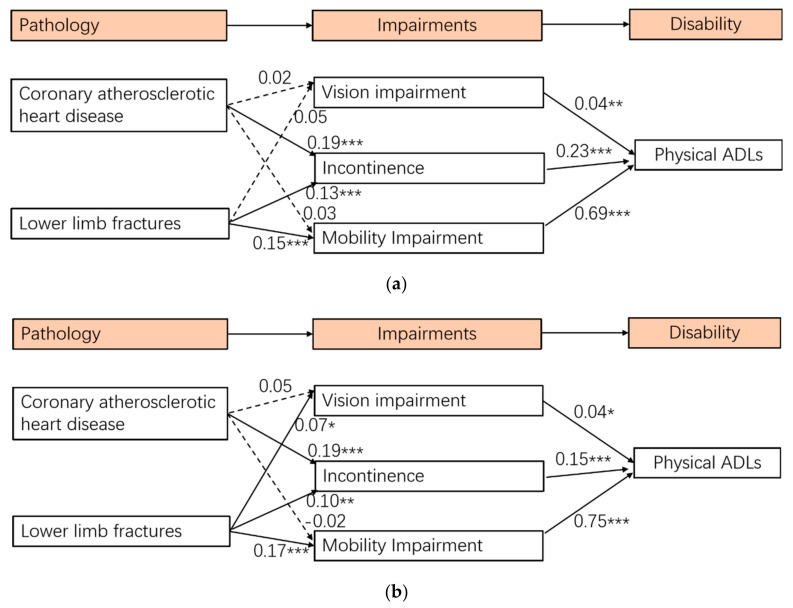
(**a**) Multigroup analysis in the SEM among women. (**b**) Multigroup analysis in the SEM among men. Notes: * *p* < 0.05, ** *p* < 0.01, *** *p* < 0.001; dashed lines represent non-significant pathways. Controlled age, education, and marital status.

After identifying a well-fitted measurement model, the relationships between all variables in the structural model were tested. The results of the structural model show a good fit for the data (Chi-square/df = 3.79, *p* < 0.001, CFI = 0.989, TLI = 0.983, RMSEA = 0.033, and SRMR = 0.020) (see Figure 1). The results of the structural model show that coronary atherosclerotic heart disease had a significant influence on incontinence, while it was not statistically significant in predicting vision impairment and mobility impairment. Lower limb fractures had significant effects on vision impairment, incontinence, and mobility impairment. Moreover, vision impairment, incontinence, and mobility impairment had significant effects on physical ADLs. Three SEMs (Figure 2a–c) revealed that coronary atherosclerotic heart disease was significantly associated with mobility impairment among older adults aged 75–84. Among older adults aged 85 and over, lower limb fractures were significantly associated with vision impairment, while vision impairment was not significantly associated with physical ADLs. Two SEMs (Figure 3a,b) revealed that lower limb fractures were significantly associated with vision impairment among men, while this association was not significant among women.

## 4. Discussion

We identified multimorbidity patterns and explored the disablement process in public long-term care insurance claimants in Chinese older adult population. In general, our results support the Disablement Process model in a sample of Chinese long-term care insurance claimants. An important contribution of this research to the disability literature is the addition of multimorbidity patterns to the disablement model.

Using LCA techniques, three multimorbidity patterns were identified in the population: coronary atherosclerotic heart disease, lower limb fractures, and other diseases. Previous research using the same techniques has identified five multimorbidity patterns among the civilian noninstitutionalized US population: the healthy group, the hypertensive group, the respiratory conditions group, the heart disease group, and the severely impaired group [13]. Such discrepancies may be due to the population differences, as our study population included potential physically disabled older people. To our knowledge, this was the first study to explore multimorbidity patterns among Chinese long-term care insurance claimants that indicated specific patterns of multimorbidity associated with potential physical disability.

Impairment, which was measured in this study through vision impairment, incontinence, and mobility impairment, was an intermediate step in the main pathway from multimorbidity patterns to physical ADL disability. However, different pathways were identified for specific patterns of multimorbidity. For example, coronary atherosclerotic heart disease increased incontinence and thus served to indirectly increase levels of physical ADL disability, while lower limb fractures were associated with increased vision impairment, incontinence, and mobility impairment, and thus served to indirectly increase levels of physical ADL disability. The findings are consistent with those of previous studies [13,18]. Lower limb fractures were associated with mobility impairment [8,19,20], and there was a high prevalence of incontinence in older adults with fractures [21]. Mobility impairment [22,23], incontinence [24,25], and vision impairment [26,27,28] were associated with the likelihood of disability.

Caution should be exercised in explaining the pathways within the conceptual framework and the cross-sectional design of the study. First, the pathological factor, such as muscle dysfunction [25] and brain nerve changes [29], may have contributed to the association between coronary atherosclerotic heart disease or lower limb fractures and incontinence. Second, the cross-sectional design precluded inferences on causality. For example, a significant association between lower limb fractures and vision impairment was shown in our study, and the possible explanation may be that vision impairment may increase the risk of falls and fractures in older adults [30,31,32]. Longitudinal studies are needed in the future to further evaluate the disablement model.

The findings expand our understanding of the disablement process among long-term care insurance claimants and provide further evidence for developing long-term care services and supports (LTSS). More attention should be paid to specific multimorbidity patterns, such as coronary atherosclerotic heart disease and lower limb fractures, and early screening is needed to identify and manage these chronic diseases so as to reduce the likelihood of impairments and disability [33]. Furthermore, early screening and management of impairments are also important, for example, to develop assessment tools for urinary and/or fecal incontinence [34] and to take evidence-based approaches to facilitate bowel and bladder management [35]. This can help slow down the process of ADL disablement.

When examining the disablement process among different age groups, we found that coronary atherosclerotic heart disease was significantly associated with mobility impairment among older adults aged 75–84, and lower limb fractures were significantly associated with vision impairment among older adults aged 85 and over. When examining the disablement process among men and women, we found that differences existed that lower limb fractures were significantly associated with vision impairment for men, but this association was not significant for women. This was consistent with previous research that found no significant association between visual acuity and risk of hip fracture among women [36]. However, speculating the reasons for these findings is infeasible at this stage. Future exploratory research is necessary. These results extend our knowledge of the disablement process in regards of age groups and gender differences.

This study had several strengths. It was the first study using Chinese long-term care insurance claimants’ data to examine the disablement process, and it attempted to address the lack of systematic research on the process of becoming ADL disabled among potential physically disabled people. In addition, the multimorbidity patterns of the potential physical disabled people were identified using LCA to contribute to the multimorbidity and disability research by focusing on the qualitative combinations of conditions among potential physically disabled people. Finally, the chronic condition data were collected by trained professionals (doctors) using claimants’ medical history, clinical examination, self-reported information, and proxy interviews to guarantee the reliability of data.

Several limitations should be noted. First, this was a cross-sectional study, which precluded inference on causality. Second, the list of chronic conditions was not comprehensive. As this was a secondary analysis study, we were limited to what had been collected. Therefore, the availability of the chronic condition data may have affected the resulting pattern identification [13]. Meanwhile, we only used education, rather than both income and education, as proxy for socioeconomic status due to data collection limitation. Third, the study sample comprised potential physically disabled older people and was not population-based older adults. Therefore, these findings may not be generalizable to the whole older people. Fourth, the role of external factors in the Disablement Process model was not included in this study, so the current analyses represent only a partial test of the Disablement Process model.

## 5. Conclusions

This research contributes to a better understanding of multimorbidity patterns and the disablement process among potential physically disabled older people. Our findings suggest that different impairments exist from specific patterns of multimorbidity to physical ADL disability. The results may provide insight for researchers and policy makers to develop tailored preventive and regular care and provide support for potential physically disabled older people according to their subgroups and course of disablement.

## Figures and Tables

**Table 1:** Participants’ characteristics (n = 2604).

Variable	n (%)
Age (years)	
60–74	803 (30.8)
75–84	890 (34.2)
≥85	911 (35.0)
Sex	
Female	1391 (53.4)
Male	1213 (46.6)
Education	
Illiterate	1426 (54.8)
Primary school	736 (28.2)
Middle school and more	442 (17.0)
Marital status	
Married	1935 (74.3)
Others	669 (25.7)
Number of chronic conditions	
0	673 (25.8)
1	1205 (46.3)
2	595 (22.9)
3	110 (4.2)
4	17 (0.7)
5	4 (0.1)

**Table 2:** LCA model fit statistics.

Classes	aBIC	Entropy	LMR	BLRT	Relative Class Size
2	15,771.478	0.784	<0.0001	<0.0001	28.3/71.7
3	15,763.588	0.979	<0.0001	<0.0001	19.0/26.4/54.6
4	15,784.687	0.816	0.0070	0.0732	17.3/3.0/53.1/26.6

Note: aBIC, sample-size adjusted BIC; LMR, *p*-value for the Lo–Mendell–Rubin likelihood ratio test; BLRT, *p*-value for the bootstrapped likelihood ratio test.

**Table 3:** Proportion of 2604 Long-term Care Insurance claimant aged ≥ 60 years within each latent class assignment based on disease onset.

Diseases	Coronary Atherosclerotic Heart Disease	Lower Limb Fractures	Other Diseases
Chronic obstructive pulmonary disease	12	5	10
Chronic pneumonia	6	4	7
Parkinson’s disease	7	6	8
Diabetes	2	1	2
Cerebral hemorrhage	3	3	4
Hypertension	1	1	3
Advanced cancer	11	27	19
Coronary atherosclerotic heart disease	100	3	0
Cerebral infarction	5	4	14
Lower limb fractures	15	100	0
Percentage of cohort	19.0	26.4	54.6

**Table 4:** Confirmatory factor analysis and factor loadings.

Five Factors and Scale Items	Standardized Loading
Mobility	
turning over in the lying position	0.845
from sitting to standing	0.921
keep sitting in a chair	0.866
walk (move) about 5 m on the flat floor	0.854
keep balance	0.825
upstairs and downstairs	0.747
Incontinence	
urinary incontinence	0.957
fecal incontinence	0.941
Physical ADLs	
eating	0.891
brushing	0.792
washing	0.803
grooming	0.743
wearing clothes	0.846
wearing pants	0.870
bathing	0.763
toileting	0.806

Notes: All standardized factor loadings are significant at *p* < 0.001.

## Data Availability

The data that support the findings of this study are available from the corresponding author, Y.L., upon reasonable request.

## Supplementary Materials

The following supporting information can be downloaded at: https://www.mdpi.com/article/10.3390/ijerph19020645/s1, File S1.
