# Supplementary material for: Multimorbidity Patterns and the Disablement Process among Public Long-Term Care Insurance Claimants in the City of Yiwu (Zhejiang Province, China)
Source: Int J Environ Res Public Health. 2022 Jan 6;19(2):645. doi: 10.3390/ijerph19020645 (PMC8775810; doi:10.3390/ijerph19020645)
Supplement: Supplementary file 1 [file ijerph-19-00645-s001.zip › ijerph-1470437-supplementary.pdf]

International Journal of
Environmental Research and Public Health

MDPI

Supplementary file 1. Scales of impairment and disability assessment- Multimorbidity Patterns and the Disablement Process among Public Long-term Care Insurance Claimants in the City of Yiwu (Zhejiang Province, China)

Please read the following statements carefully and determine the one best describes the situation.

Vision impairment

1. *Which statement best describes your present vision?*

1=basically normal;
2=a little vision impairment and some difficulty in activities of daily living;
3=some vision impairment and severe difficulty in activities of daily living;
4=severe impairment: only with a little visual perception (e.g., perception of the shape of a hand within 1 m);
5=severe impairment: blindness or no visual perception.

Mobility impairment

1. *Turning over in the lying position*

1 = without assistance;
2 = needs verbal cueing or supervision only;
3 = limited hands-on assistance;
4 = extensive hands-on assistance;
5 = with fully assistance

2. *From sitting to standing*

1 = without assistance;
2 = needs verbal cueing or supervision only;
3 = limited hands-on assistance;
4 = extensive hands-on assistance;
5 = with fully assistance

3. *Keep sitting in a chair*

1 = without assistance;
2 = needs verbal cueing or supervision only;
3 = relying on or grasping a fixed object;
4 = relying on a fixed object and needs supervision;
5 = with fully assistance

4. *Walk (move) about 5 meters on the flat floor*

1 = without assistance;
2 = needs a cane or walking aid;
3 = needs verbal cueing or wheelchair;
4 = limited hands-on assistance;
5 = extensive hands-on assistance or with fully assistance

5. *Keep balance*

1 = without assistance;
2 = needs verbal cueing or supervision only;

3 = relying on or grasping a fixed object;
4 = relying on a fixed object and needs supervision;
5 = with fully assistance

6. Upstairs and downstairs

1 = without assistance;
2 = needs verbal cueing or supervision only;
3 = limited hands-on assistance;
4 = extensive hands-on assistance;
5 = with fully assistance

Incontinence

1. Which statement best describes your present urinary incontinence?

1=without incontinence;
2=about once a month;
3=about once a week;
4 = about once per day;
5=almost every time.

2. Which statement best describes your present fecal incontinence?

1=without incontinence;
2=about once a month;
3=about once a week;
4 = about once per day;
5=almost every time.

Physical ADLs

1. eating

1 = without assistance;
2 = needs verbal cueing or supervision only;
3 = limited hands-on assistance;
4 = extensive hands-on assistance;
5 = with fully assistance

2. brushing

1 = without assistance;
2 = needs verbal cueing or supervision only;
3 = limited hands-on assistance;
4 = extensive hands-on assistance;
5 = with fully assistance

3. washing

1 = without assistance;
2 = needs verbal cueing or supervision only;
3 = limited hands-on assistance;
4 = extensive hands-on assistance;
5 = with fully assistance

4. grooming

1 = without assistance;
2 = needs verbal cueing or supervision only;
3 = limited hands-on assistance;
4 = extensive hands-on assistance;
5 = with fully assistance

5. wearing clothes

1 = without assistance;
2 = needs verbal cueing or supervision only;
3 = limited hands-on assistance;
4 = extensive hands-on assistance;
5 = with fully assistance

6. wearing pants

1 = without assistance;
2 = needs verbal cueing or supervision only;
3 = limited hands-on assistance;
4 = extensive hands-on assistance;
5 = with fully assistance

7. bathing

1 = without assistance;
2 = needs verbal cueing or supervision only;
3 = limited hands-on assistance;
4 = extensive hands-on assistance;
5 = with fully assistance

8. toileting

1 = without assistance;
2 = needs verbal cueing or supervision only;
3 = limited hands-on assistance;
4 = extensive hands-on assistance;
5 = with fully assistance
